# JaalTaka: A benchmark dataset for detecting counterfeit Bangladeshi banknotes

**DOI:** 10.1016/j.dib.2025.112377

**Published:** 2025-12-11

**Authors:** Bibhas Roy Chowdhury Piyas, Fatama Jannat Tisha, Sadia Rahman, Shahrin Islam, Bijoy Roy Chowdhury Preenon

**Affiliations:** a Department of Software Engineering, Daffodil International University, Dhaka 1216, Bangladesh; b Department of Computer Science and Engineering, Chittagong University of Engineering and Technology, Chattogram 4349, Bangladesh; c Department of Electrical and Electronic Engineering, Independent University, Dhaka 1229, Bangladesh

**Keywords:** Counterfeit banknote, Banknote authentication, Bangladeshi Banknote, Multi-view imaging, Deep learning

## Abstract

The rapid spread of counterfeit currency threatens global economic stability by undermining public confidence and distorting financial systems. These challenges are particularly severe in cash-dependent economies, where reduced trust in paper currency limits transactions and hinders financial inclusion. Although substantial research exists on banknote recognition and classification, studies specifically targeting counterfeit detection remain limited. The challenges and barriers associated with collecting counterfeit banknotes are a key factor behind the limited resources in this field. To bridge this gap, we present a benchmark dataset named, “JaalTaka”, consisting of 1390 images of Bangladeshi banknotes, including 802 real and 588 counterfeit notes. Due to the subtle differences in security features between real and fake banknotes, six separate images of different segments were captured for each note to highlight these critical elements. Genuine banknotes were obtained from three national banks in Bangladesh, while counterfeit notes were sourced from the Rapid Action Battalion (RAB), a specialized security unit, for research purposes. To ensure robustness, the dataset includes banknotes in diverse conditions including, new, worn, user-marked and stained. This is the first publicly available dataset providing a reliable foundation for developing effective Bangladeshi counterfeit banknote detection systems. The dataset can serve as a benchmark resource for research on counterfeit banknote detection and improving financial security.

Specifications Table

**Table:** 

Subject	Computer Science
Specific subject area	Counterfeit banknote detection, Banknote authentication, Image classification, Deep learning, Computer Vision
Type of data	Image
Data collection	The dataset, “JaalTaka” is developed by capturing images of both the front and back sides of real and counterfeit banknotes using a smartphone camera. To highlight subtle differences in security features, six separate images of different segments were taken for each banknote. Genuine notes were sourced from three government banks in Bangladesh, while counterfeit notes were obtained from the Rapid Action Battalion (RAB), a specialized security unit of Bangladesh, through formal collaboration for research purposes. To ensure robustness, the dataset includes banknotes in various physical conditions, including new, old, stained, torn, and graffiti-covered, making it suitable for real-world scenarios.
Data source location	Genuine banknotes were sourced from three government banks of Bangladesh - Sonali Bank Ltd, Agrani Bank Ltd, and Rupali Bank Ltd, while counterfeit notes were obtained from the Rapid Action Battalion (RAB), a specialized security unit of Bangladesh, through formal collaboration for research purposes. Access to counterfeit notes was granted under official supervision, allowing our team to capture the necessary images in a secure and controlled environment with the cooperation of the authorities.
Data accessibility	Repository name: Mendeley Data Data identification number: 10.17632/2m7wk5cy4c.2 Direct URL to data: https://data.mendeley.com/datasets/2m7wk5cy4c/2
Related research article	None

## 1 Value of the Data

• This is the first publicly available dataset that serves as a reliable foundation for developing robust and effective Bangladeshi banknote authentication systems.
• The dataset features banknotes in diverse conditions, including new, worn, user-marked and stained enhancing its applicability in real-world scenarios.
• Multiple images of each banknote capturing different segments emphasize subtle security features and enable detailed analysis with precise extraction for accurate counterfeit detection.
• Our dataset supports the development of automated systems that reduce reliance on manual inspections in high-volume cash-handling environments. It can also be applied in ATMs and vending machines to make transactions safer and more reliable.
• This dataset fills a critical resource gap for researchers in monetary integrity and counterfeit detection and establishes a benchmark for subsequent studies.

## 2 Background

Counterfeit banknotes continue to pose a global challenge, while publicly available and well-annotated datasets for banknote authentication remain scarce. However, several studies have addressed the detection and classification of banknotes from different countries. Baek et al. [1] introduced multispectral banknote datasets, scanning each banknote at 50 DPI across six wavelengths (400–1000 nm), primarily focusing on spectral analysis. Authors of [2] proposed a dataset of Bangladeshi 500 and 1000 BDT banknotes for counterfeit detection, but it includes only genuine notes. Dittimi et al. [3] developed a Nigerian Naira dataset comprising 2310 genuine and 2048 counterfeit notes, collected from various regions with denominations ranging from Five to One Thousand Naira, though it lacks detailed segment-level imagery highlighting security features . Study [4] designed a Euro dataset covering 10€, 20€ and 50€ banknotes captured under varying luminance conditions and study [5] presented a comprehensive dataset of Taiwanese banknotes without counterfeit samples. Authors of [6] developed the Indian coin dataset (CIDCIC), which covers seven denominations. Meshram et al. [7] included 10 and 5 denominations of Indian and Thai banknotes, but they didn’t consider variation in note condition. Study [8] introduced NSTU-BDTAKA for all Bangladeshi currency recognition. Authors of [9] covered Kazakhstan banknotes, while [10] provided annotated Ghanaian coins and banknotes. Lastly, [11] offered 9315 images of old and new Peruvian notes where all of them were genuine. “JaalTaka” addresses this gap by providing a publicly available dataset of genuine and counterfeit Bangladeshi banknotes.

## 3 Data Description

Counterfeit currency detection is crucial for maintaining the integrity of cash-based economies, as counterfeit notes distort the money supply, disrupt pricing, erode public trust, and result in operational losses for banks and retailers. To enable the automated identification of counterfeit banknotes, constructing a diverse and representative dataset is essential. In this work, to develop a diverse dataset, we have included denominations of 500 and 1000 banknotes for both practical and methodological reasons. Higher-denomination notes are the prime targets for counterfeiters since they maximize illicit returns per transaction while justifying the cost of sophisticated forgery techniques such as specialty inks, watermarks and micro-lettering. These denominations are also widely circulated in everyday cash transactions which makes the dataset directly relevant to real-world contexts. Furthermore, their security features are highly comparable, which enhances the dataset’s generalizability.

### 3.1 Security parameters of Bangladeshi banknotes

**Fig. 1:**
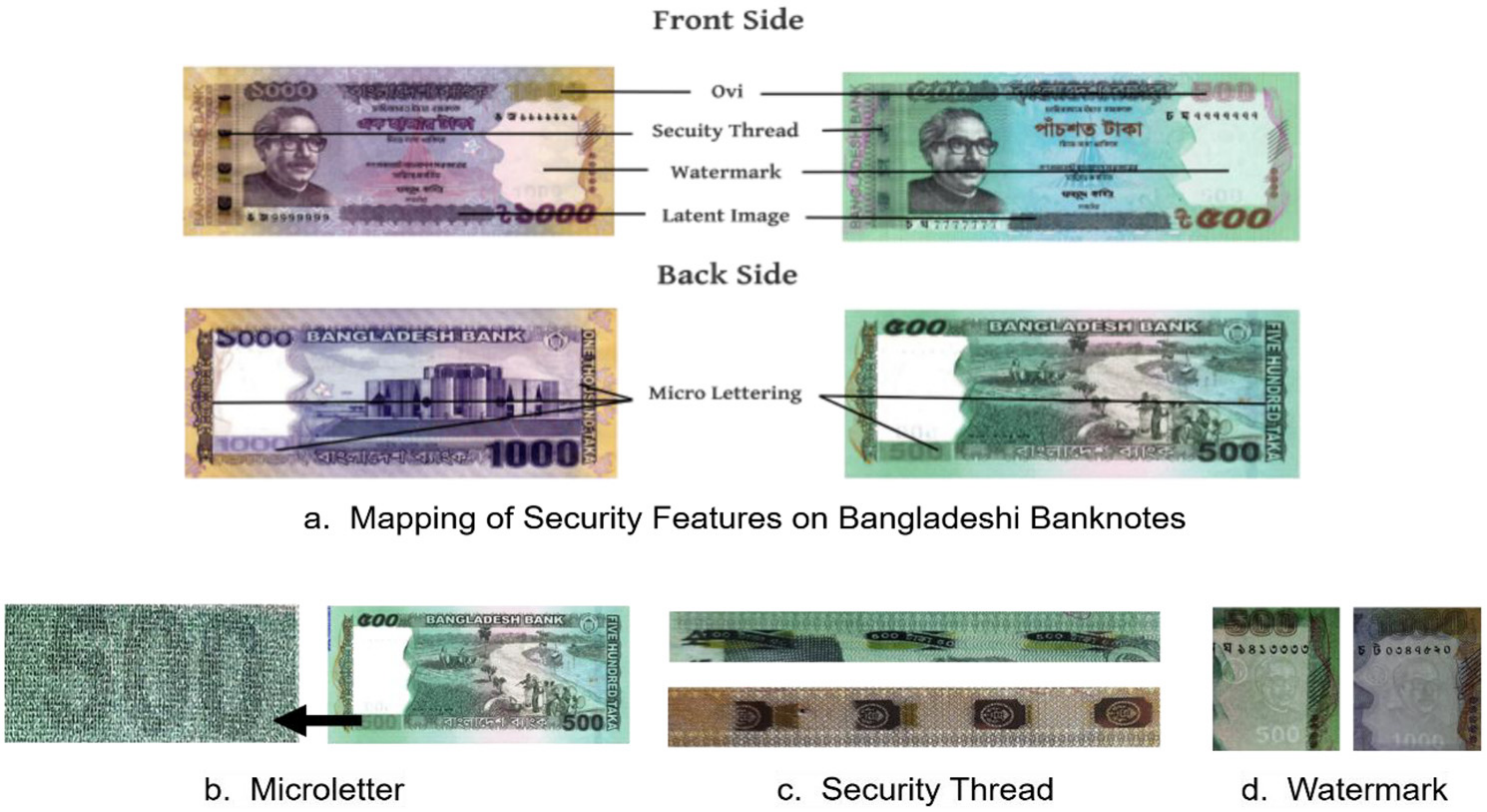
Security parameters of Bangladeshi banknotes.

In our study, we examined the security features of Bangladeshi banknotes of Tk. 500 and Tk. 1000. Key features include micro-letters that include repeated prints of "BANGLADESH BANK" and the note denomination, which are difficult to replicate and can be observed with an inspection lens. A portrait of Bangabandhu Sheikh Mujibur Rahman along with an embedded electrolyte mark indicating its denomination, offers a crucial visual security element. A security thread of 4 mm width, embedding the Bangladesh Bank logo and note value, appears on the left side of the notes. Other features include intaglio ink with raised patterns and text for tactile recognition, a latent image showing the denomination when held horizontally, optically variable ink (OVI) changing colour with viewing angle, and an iridescent stripe on the Tk. 1000 note that shifts colour under slight oscillation. Together, these features make the notes highly secure against counterfeiting. For this dataset, the focus was on security features that can be visually verified and are suitable for authentication using standard camera devices. Advanced features such as infrared or ultraviolet patterns and machine-readable codes were excluded due to restricted access, legal limitations and the need for specialized equipment. Including such features would make the dataset less practical for real-world applications where simple, camera-based authentication is preferred. Fig. 1 illustrates the various security features of Bangladeshi banknotes.

### 3.2 Data acquisition sources

Data acquisition was the most challenging aspect of this work, particularly in obtaining counterfeit banknotes due to their limited availability and the associated security and accessibility constraints. Genuine banknotes were collected without difficulty from several government banks of Bangladesh - Sonali Bank Ltd., Agrani Bank Ltd., and Rupali Bank Ltd. Counterfeit banknotes were obtained through formal collaboration with the Rapid Action Battalion (RAB), a specialized security unit of Bangladesh, for research purposes. Access was granted under official supervision, enabling our team to capture images of counterfeit notes in a secure and controlled setting with the cooperation of the authorities. All collected banknotes were systematically labelled as either real or fake to ensure reliable dataset construction. Table 1 provides detailed information about the sources of data collection.

**Table 1:** Data acquisition sources.

Banknote Type	Source	Source Type	Location
Real	Sonali Bank Ltd.	Government Bank	Joydebpur, Gazipur
	Agrani Bank Ltd.	Government Bank	Joydebpur, Gazipur
	Rupali Bank Ltd.	Government Bank	Joydebpur, Gazipur
Counterfeit	RAB	Special Security Force	Mirpur 1, Dhaka

### 3.3 Dataset visualization

The security features of Bangladeshi banknotes are distributed across both the front and back sides. Many of these features are highly subtle, which makes it difficult to distinguish genuine notes from counterfeits with a single image. To capture these intricate details, multiple images from different regions of each banknote are necessary. In this dataset, six images of various segments of a banknote were collected to highlight the most significant security features. This approach ensures that the dataset is particularly valuable for multi-view models, where the final prediction can be derived from the combined analysis of all six views. Table 2 presents example banknotes with its segmented images which illustrates the image acquisition process employed in developing the dataset.

**Table 2:** Visualization of the multi-view capture process of banknotes.

### 3.4 Variability in physical condition in banknotes

To make the dataset more robust, we captured banknotes in a variety of physical conditions. The genuine notes displayed significant natural variability resulting from real-world circulation, including wear and tear, stains, user markings, graffiti, and repair artifacts such as tape or staple marks. In contrast, counterfeit notes exhibited much less variation, as they typically do not circulate widely in the market and are often available only in relatively new condition. Moreover, counterfeit producers tend to replicate similar patterns, which leads to limited diversity across forged notes. This shows that while counterfeit notes are mostly uniform, the genuine notes offer considerable variability, making the dataset more realistic and useful.

The genuine banknotes used in this study were sorted according to their physical condition to ensure that the dataset reflects real-world circulation scenarios. Each note was manually examined and classified into four distinct deterioration categories based on visible wear and external alterations:

• **New:** crisp and less circulated notes with no visible wear or folds.
• **Old:** visibly worn notes with folds, fading, or surface wear from extended circulation.
• **User marking:** notes containing handwritten text, signatures or pen marks added by users.
• **Stains:** notes exhibiting discoloration or marks caused by liquids, dirt, or other staining agents.

**Fig. 2:**
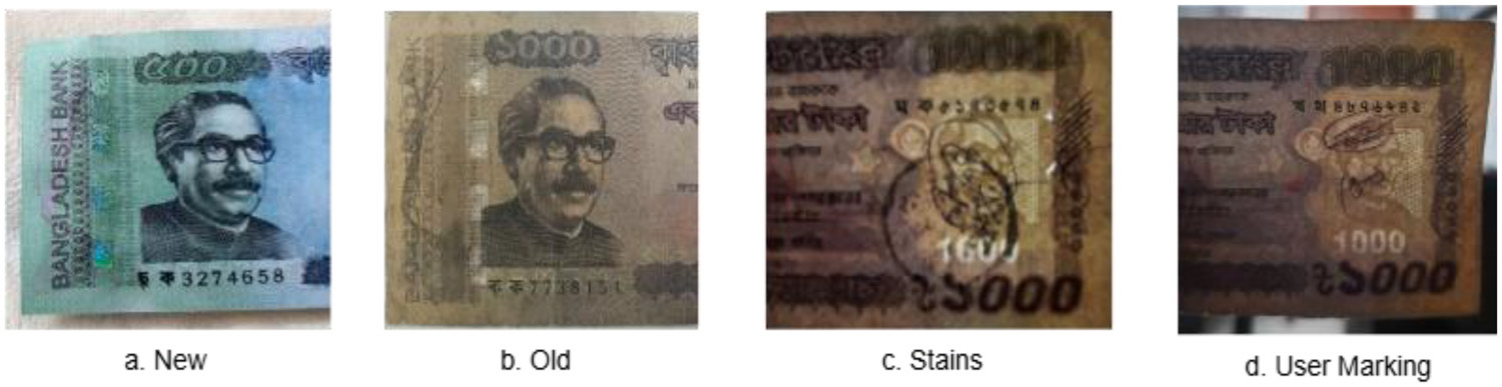
Different physical states of banknotes.

Fig. 2 illustrates examples of these variations in the collected banknotes.

### 3.5 Data distribution

**Fig. 3:**
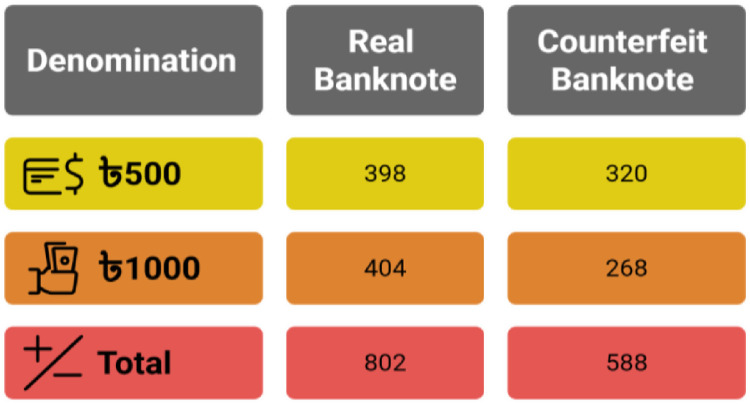
Dataset distribution analysis.

The dataset contains 1390 banknotes with 802 real and 588 counterfeit. In the real set, 398 images are of Tk. 500 notes and 404 of Tk. 1000 notes. In the counterfeit set, 320 images are of Tk. 500 notes and 268 of Tk. 1000 notes. Fig. 3 shows the dataset distribution by class and denomination.

Table 3 presents the dataset volume, showing the number of sample images across classes along with their corresponding storage sizes.

**Table 3:** Dataset sample count and storage.

Banknote Type	Number of notes	Number of Images	Total Size
Real	802	4812	4.24 GB
Counterfeit	588	3528	2.86 GB
Total	1390	8340	7.1 GB

## 4 Experimental Design, Materials and Methods

To enable automatic detection of counterfeit banknotes, this paper developed a diverse dataset comprising both real and fake banknotes. To capture subtle details of the security features and improve the reliability of authentication, six images of different segments of each banknote were collected. Genuine notes were acquired from three government banks in Bangladesh without difficulty. On the other hand, due to limited availability and strict security restrictions, counterfeit banknotes were obtained through formal collaboration with the Rapid Action Battalion (RAB), a specialized security unit of Bangladesh. Access was granted for research purposes under official supervision, which allowed our team to take images of counterfeit notes under a secure and controlled environment with the cooperation of the authorities.

All notes were systematically labelled as either real or fake to guarantee the reliability of the dataset construction. All Images were saved in .jpg format. To make it robust, the dataset includes banknotes in varied physical conditions such as wear and tear, stains, user markings, graffiti, and repair artifacts (e.g., tape or staple marks). Image acquisition was performed using smartphone cameras, which provided sufficient resolution to capture subtle security features and ensured cost-effectiveness, portability, and reproducibility. This choice also reflects real-world scenarios where mobile-based solutions are particularly suited for counterfeit banknote detection.

**Fig. 4:**
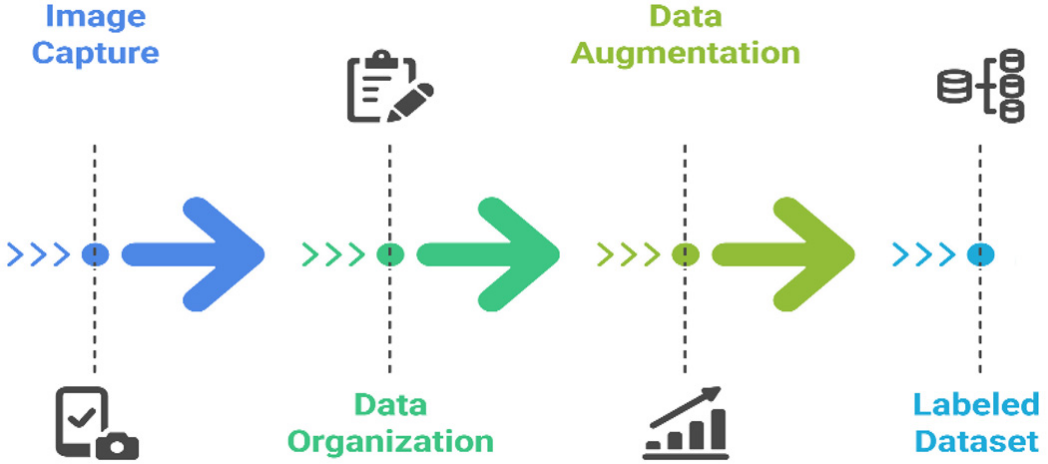
Dataset development workflow.

The dataset is publicly released under the name JaalTaka and organized into two subfolders: real_notes and fake_notes. Each subfolder contains sequentially labelled directories for individual notes (e.g., note_001, note_002). Each note directory contains six segment images (e.g., note_001_1, note_002_2). To further enhance robustness, several data augmentation techniques were applied, including rotation, Gaussian noise addition, brightness–contrast enhancement, and combined rotation with brightness contrast enhancement. To construct the dataset, we followed a systematic methodology, illustrated in Fig. 4, that details each stage from data acquisition to final dataset creation.

### 4.1 Image acquisition setup

Smartphone cameras were used for image acquisition as they provide sufficient resolution to capture subtle security features and ensure cost-effectiveness, portability, and reproducibility. This choice also aligns with real-world deployment scenarios where mobile-based solutions are practical for counterfeit detection. All images were saved as JPEG (.jpg). In this task, 4 smartphone models were used - Samsung Galaxy A50, Samsung Galaxy M31, Redmi Note 8, and Redmi Note 9 Pro during dataset acquisition. Table 4 shows the camera specifications of the smartphone models used for image acquisition.

**Table 4:** Camera specifications for capturing banknotes.

Mobile Model	Samsung Galaxy A50	Samsung Galaxy M31	Redmi Note 8	Redmi Note 9 Pro
Aperture	f/1.7	f/1.8	f/1.8	f/1.9
Focal Length	4mm	5mm	4mm	5mm
Metering Mode	Spot	Spot	Center-weighted average	Center-weighted average

### 4.2 Dataset directory

**Fig. 5:**
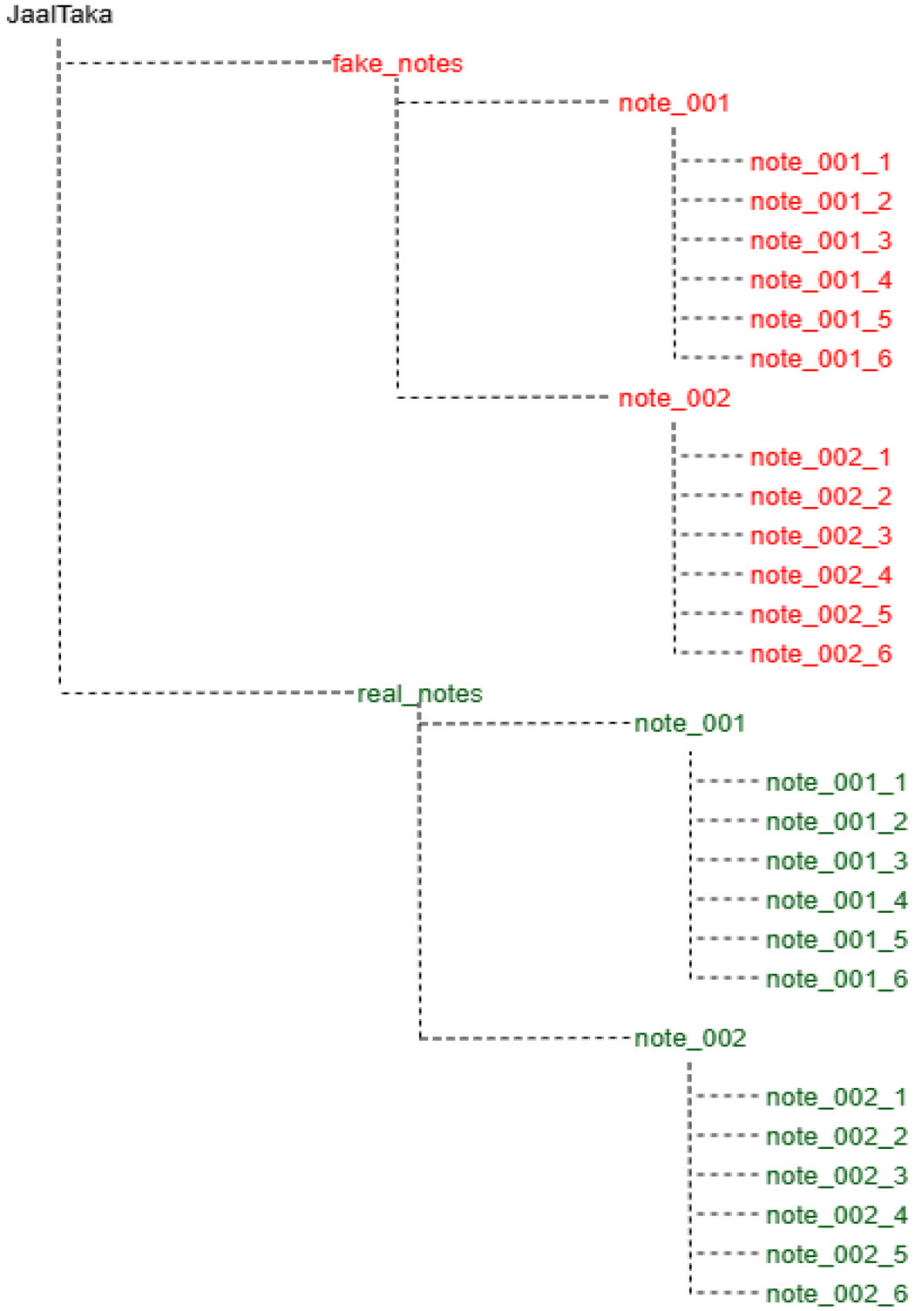
Dataset directory structure.

The dataset is released as a single folder named “JaalTaka”, which contains two subfolders: real notes and fake notes. Each of these subfolders is further organized into separate folders for individual banknotes, labelled sequentially as note_001, note_002, and so on. Within each note folder, six images are stored corresponding to different segments of the same banknote. For example, the folder note_001 contains files named note_001_1.jpg, note_001_2.jpg,…,note_001_6.jpg. Fig. 5 illustrates the directory structure of the dataset

### 4.3 Data augmentation

In real-world scenarios, banknotes may appear under varying lighting conditions, angles, rotations, or partial occlusions. Additionally, the number of collected counterfeit banknotes is limited due to restricted access. As discussed earlier, counterfeit notes exhibit much less variation because they rarely circulate widely and are typically available in relatively new condition. Counterfeit producers also tend to replicate similar patterns, resulting in limited diversity across forged notes.

**Fig. 6:**
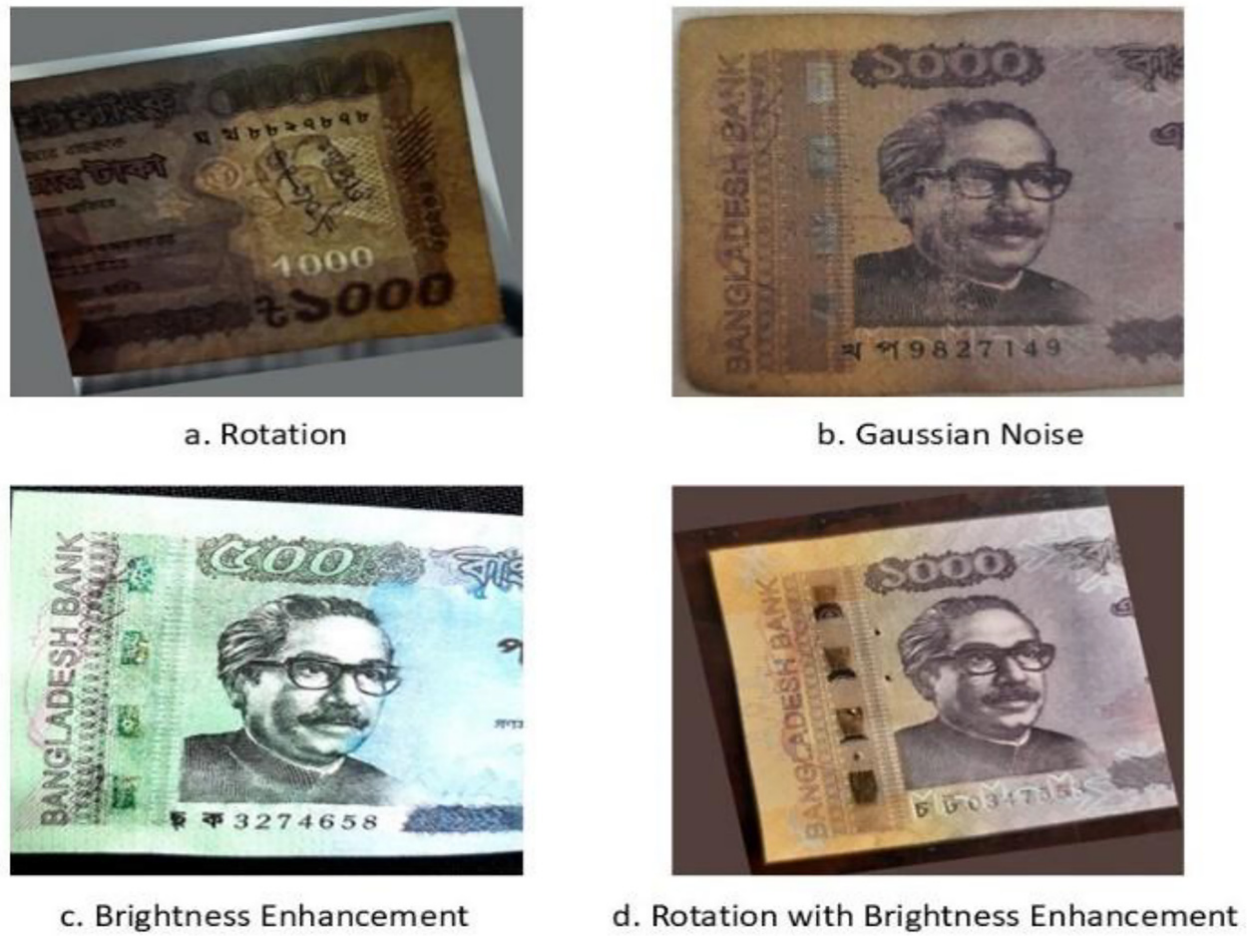
Data augmentation techniques.

To address these challenges, data augmentation was applied to increase the variability of images and expand the overall dataset. Initially, a total of 649 banknotes were collected during the investigation, including 502 genuine and 147 counterfeit notes in their original condition. We then applied augmentation techniques including rotation, Gaussian noise addition, brightness-contrast enhancement, and combined rotation with brightness-contrast enhancement. After augmentation, the dataset consists of a total of 1390 images, comprising 802 genuine and 588 counterfeit notes. Fig. 6 illustrates the various types of augmentations applied to our dataset.

Table 5 provides a clear breakdown of the collected and augmented samples by denomination and class

**Table 5:** Breakdown of collected and augmented samples by denomination and class.

Denomination	Collected Genuine	Collected Counterfeit	Augmented Genuine	Augmented Counterfeit	Total Genuine	Total Counterfeit
500 BDT	270	82	128	238	398	320
1000 BDT	232	65	172	203	404	268

### 4.4 Application of the dataset

**Fig. 7:**
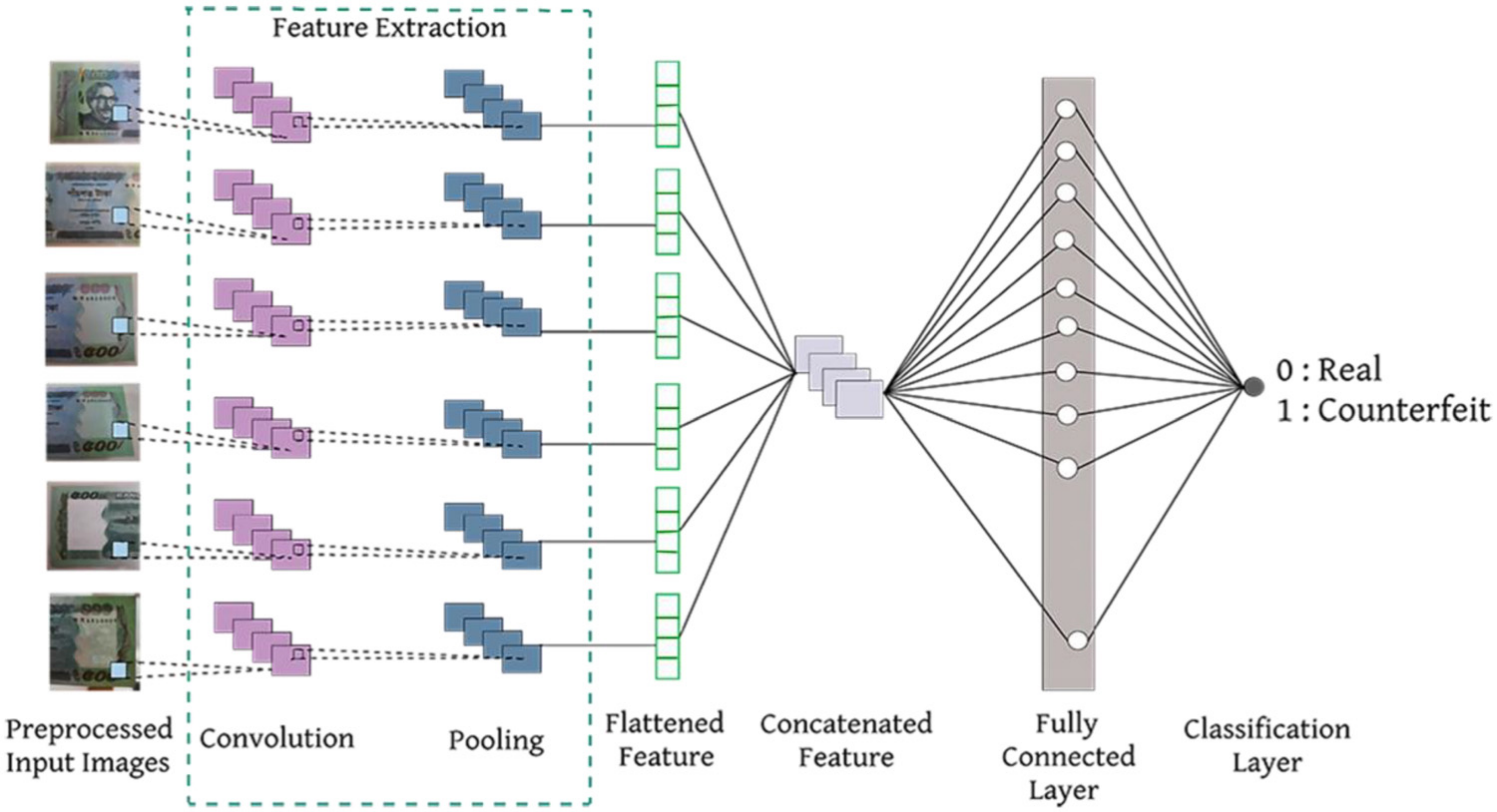
Overview of the proposed architecture.

Our developed dataset, JaalTaka, is particularly valuable for multi-view learning models, where final predictions are derived from the combined analysis of all six views. Fig. 6 provides an overview of how the dataset can be effectively utilized in a deep learning–based multi-view framework (Fig. 7).

To evaluate the dataset, several pretrained CNN architectures were explored, including ResNet50, InceptionV3, DenseNet121, VGG16, and MobileNetV2 for counterfeit banknote detection. The experimental results across various performance metrics are summarized in Table 6.

**Table 6:** Performance comparison with different models.

Models	Accuracy	Precision	Recall	F1 Score
ResNet50	.985	.98	.98	.98
InceptionV3	.974	.97	.97	.97
DenseNet121*	**.989**	**.99**	**.98**	**.99**
VGG16	.982	.98	.97	.98
MobileNetV2	.982	.98	.98	.98

## Limitations

While we have made our best effort to develop a comprehensive dataset for the automatic detection of Bangladeshi counterfeit banknotes, this work has certain limitations. The dataset includes only the banknotes of 500 BDT and 1000 BDT denominations for a few practical and methodological reasons. Higher-denomination notes are the prime targets for counterfeiters, as they maximize illicit returns per transaction and justify the use of sophisticated forgery techniques, such as specialty inks, watermarks, and micro-lettering. Due to the limited availability of counterfeit banknotes of other denominations, these have not been included in this work. Variability in the counterfeit banknotes was introduced using data augmentation techniques, which may not fully capture the complexity of real counterfeit notes and could introduce augmentation bias. Moreover, genuine notes were collected from a limited region and counterfeit samples came from a single source due to limited availability and accessibility constraints. These factors may affect the model’s generalizability to other denominations. Future work should expand the dataset by including additional denominations, multiple sources and broader geographic coverage to enhance real-world applicability. Furthermore, we aim to expand the dataset with images captured using advanced banknote processing systems (e.g., BPS by Giesecke & Devrient) to make it more diverse, inclusive and adaptable for both smartphone-based and high-end authentication systems.

## Ethics Statement

The JaalTaka dataset has been developed following strict ethical data collection practices. All genuine banknotes were collected from authorized banks in Bangladesh, and counterfeit banknotes were obtained through formal collaboration with the Rapid Action Battalion (RAB), under official supervision for research purposes. No unauthorized access was involved in the data collection process. All data acquisition was conducted in a secure and controlled environment to ensure the safety of personnel and compliance with legal regulations. The dataset is intended solely for research purposes that focus on the development of automatic counterfeit detection systems to enhance monetary security. Care was taken to label and organize all banknotes systematically to preserve the integrity of the sources. This work does not involve any private information, and no individual was identified or targeted in the process. By following these ethical principles, our dataset enables responsible research in financial security while fully complying with legal and safety standards.

## Credit Author Statement

Bibhas Roy Chowdhury Piyas: Conceptualization, Data curation, Methodology, Software, Writing – original draft, Visualization. Fatama Jannat Tisha: Conceptualization, Data curation, Methodology, Software, Writing – original draft, Visualization. Sadia Rahman: Data curation, Methodology, Investigation, Validation, Writing – review & editing. Shahrin Islam: Data curation, Investigation, Validation, Writing – review & editing. Bijoy Roy Chowdhury Preenon: Data curation, Validation, Writing – review & editing.

## Declaration of Competing Interest

The authors declare that they have no financial or personal affiliations that might have affected the research reported in this study.

## Data Availability

Mendeley DataJaalTaka: A Benchmark Dataset for Detecting Counterfeit Bangladeshi Banknotes (Original data).
